# Translation, Cultural Adaptation and Validation of the Nurses Self-Concept Instrument (NSCI) to Spanish

**DOI:** 10.3390/ijerph20021529

**Published:** 2023-01-14

**Authors:** Alba Laborería-Romances, Carlos Navas-Ferrer, Ana Anguas-Gracia, Marta Callén-Galindo, Isabel Antón-Solanas, Fernando Urcola-Pardo

**Affiliations:** 1 Hospital Clínico Universitario Lozano Blesa, 50009 Zaragoza, Spain; 2 Department of Physiatry and Nursing, Faculty of Health Sciences, University of Zaragoza, 50009 Zaragoza, Spain; 3 Water and Environmental Health Research Group (DGA-B43-20R), 50009 Zaragoza, Spain; 4 Research Group in Care (GIIS081), Institute for Health Research Aragón, University Clinical Hospital Lozano Blesa, 50009 Zaragoza, Spain; 5 Hospital San Jorge, Avda. Martínez de Velasco 6, 22005 Huesca, Spain; 6 Research Group Nursing Research in Primary Care in Aragón (GENIAPA) (GIIS094), Institute of Research of Aragón, 50009 Zaragoza, Spain

**Keywords:** self-concept, leadership, nurses, validation, Spanish

## Abstract

Professional self-concept in nurses is understood as the way nurses think and feel about themselves in their nursing role and is both a predictor of quality of care and a protective factor against burnout. The aim of this study was to translate, culturally adapt and validate the Spanish version of the Nurses Self-Concept Instrument in a sample of 483 Spanish registered nurses. In addition, we analyzed gender differences in its dimensions in the same sample. Internal reliability was evaluated using Cronbach’s Alpha, while construct validity was assessed using both exploratory and confirmatory factor analysis. The differences between groups were analyzed using the Mann-Whitney U test. Factor distribution was different from the original model. A gender gap was observed in the Nurse Thinking and Perception of Capabilities dimensions with higher values in the women group, while in the Leadership dimension, higher values were observed in the men group. While the Spanish version of the Nurses Self-Concept Instrument is a valid and reliable tool to measure this construct, the differences in its dimensions lead to a deeper understanding of the cultural differences in the construction of professional self-concept.

## 1. Introduction

Professional self-concept in nursing is the way in which nurses think and feel about themselves in their role as nurses [1,2], and it is related to the quality of nursing care. According to Sasat et al. [2], nurses with a healthy self-concept are more competent and committed not only to their tasks’ performance but also to the people around them (colleagues or patients), thus promoting a positive work environment. Therefore, nurses with a good professional self-concept are likely to provide better patient care than those with a poor professional self-concept [3,4], improving person-centered care and patient safety activities [5]. However, nurses’ self-concept is not a fixed concept as it is deeply influenced by significant individuals and factors such as nursing’s social image, the environment and values in the workplace and their education and training, both during their nursing studies and, later on, throughout their professional career [6,7]. Specifically, environmental, social and educational conditions are likely to exert a deep influence on nurses’ self-concept, affecting their work identity [8]. Moreover, according to the literature [9,10], they may contribute to widening the gender gap within the profession, maintaining gender prejudices and leading to an imbalance in the distribution of management positions [11,12].

### Background

Self-concept is defined as the image that a person has of her or himself [13]. It is considered a hierarchical and multidimensional structure, indicating how people think and feel. It can be structured in several domains (academic, personal, physical and social). Subsequently, each domain can be divided into more specific dimensions [14]. These domains are interlinked and contribute to explaining how social gender stereotypes affect the construction of professional self-concept in healthcare workforces, and perpetuate existing gaps in gender leadership [15]. Self-concept development begins during childhood and it is further influenced and shaped by individual characteristics, life experiences, significant others and the environment, throughout life [16]. A positive self-concept is at the base of good personal, social and professional performance [17], having a deep impact on personal development and social behavior [18].

In nursing, self-concept has been associated with important aspects including the development of clinical competencies [19], quality of care, job satisfaction and professional commitment [20]. In addition, having a positive self-concept is a protective factor against burnout [21]. Moreover, nurses who have a positive professional identity tend to be more efficient in their own emotional labor, thus preventing emotional exhaustion [22].

Over the past thirty years, different instruments have been developed to measure professional self-concept in nurses. The first one, developed by Porter & Porter, was the Porter Nursing Image Scale (PNIS). This tool comprised 32 items distributed into three dimensions (interpersonal power, interpersonal relations and interpersonal ability) [23]. Subsequently, the Professional Self-Concept of Nurses Instrumentation (PSCNI) was designed by Arthur [24]. In its original version, the PSCNI comprised 56 items classified into 7 dimensions; in 1998, Arthur & Thorne designed an abridged version including 27 items classified into 5 dimensions (flexibility, skills, leadership, communication and satisfaction) [25]. The Nurses Self-Concept Questionnaire (NCSQ) comprised 36 items distributed into six dimensions (care, leadership, communication, knowledge, staff relations and nurse general self-concept) [26]. Based on these three instruments, Angel et al. [1] developed the Nurses Self-Concept Instrument (NSCI) to measure nurses’ self-concept in Australia, reducing the number of items to 14 and classifying them into four dimensions, namely care, knowledge, staff relations and leadership. This last instrument was selected to be translated and culturally adapted to Spanish as not only it was the most up-to-date version, but also it was briefer than the previous three tools [27].

Social and cultural environments where nurses train and work have a deep influence on the process of self-concept development and the conception of care [28]. In addition, this process is heavily influenced by local health policy. For example, whilst there is a lack of trained nurses in Australia, many Spanish nurses are forced to move abroad by the time they qualify [29]. These circumstances create a significant economic and job satisfaction gap among these professionals. Further, according to the EUSPERT report, Australian nurses earn 10,000 USD more per year—adjusted by purchasing power parity (PPP)—than their Spanish colleagues. Moreover, Spanish nurses have the lowest work satisfaction rate across Europe, especially in terms of work schedules and career opportunities [30]. Furthermore, the nurses who stay in Spain must face some factors which have a negative influence on their professional performance, including a lack of human and material resources and poor professional recognition. These factors can negatively affect nurses’ professional self-concept and, therefore, decrease the quality of patient care [4,31].

An early and frequent assessment of nurses’ self-concept may be useful to design and implement tools and strategies to maintain and improve professional self-concept and identity, improving the nurses’ professional performance and job satisfaction. To achieve this, it is important to identify the right tool to measure this construct in this population. Angel et al.’s NSCI [1] was validated to measure self-concept in a sample of Australian student nurses; it has also been translated and validated into Taiwanese [32] in a sample of Taiwanese students. However, to our knowledge, there have been no previous attempts to validate this tool in a population of qualified nurses. As there is also a lack of tools available to measure nurses’ professional self-concept in Spain, the aim of this study was to translate, culturally adapt and validate the NSCI in a sample of graduate nurses from Spain.

## 2. Materials and Methods

### 2.1. Study Design and Participants

The study design for the translation, cross-cultural adaptation and validation of the NSCI was divided into two phases: (1) translation and cross-cultural adaptation of the NSCI and (2) validation of the NSCI in a sample of graduate nurses.

### 2.2. Participants

Graduate Spanish nurses from two large, publicly funded, tertiary hospitals from two different cities in the region of Aragón (Hospital San Jorge (Huesca, Spain) and Hospital Clínico Universitario Lozano Blesa (Zaragoza, Spain)) and 22 primary care centers (15 rural and 7 urban) were invited to participate in this study (N = 1387). Sample size calculation for Exploratory Factor Analysis (EFA) was based on the 5–10 subjects per item rule [33]. For this analysis, a minimum sample size of 140 subjects was estimated. For Confirmatory Factor Analysis (CFA), a sample of at least 200 subjects was estimated [34].

### 2.3. Procedure

The original version of the NSCI was developed by Angel et al. [1] to measure nurses’ self-concept. It was originally composed of 14 items classified into four dimensions: (a) Care: items 1 to 3; (b) Knowledge: items 4 to 7; (c) Staff relations: items 8 to 10; and (d) Leadership: items 11 to 14. Each item is measured on an 8-point Likert scale, from 1 (definitively false) to 8 (definitively true), and the score for each dimension is calculated by its average score. The participants also answered a brief socio-demographic questionnaire, comprising age, sex, years since qualifying, current job (hospitalization or primary care) and time worked in the current post. The researchers contacted the original creators of the NSCI [1] before the beginning of the study, obtaining their permission to work with the instrument.

#### 2.3.1. Phase 1: Translation and Cross-Cultural Adaptation of the NSCI

The instrument was first translated by two independent native translators into Spanish, obtaining the first version of the instrument in Spanish. Subsequently, two different, native translators back-translated the Spanish version into English, unifying their criteria in a new-English version. The three versions of the instrument (original English, Spanish and new English) were sent to a group of 10 experts to report any semantic or validity concerns. The experts’ group was composed of four translators, four Nursing Degree teachers (two from the University of Zaragoza and two from the University of Sevilla), a nurse manager, a clinical nurse, and a PhD student. After obtaining the final version of the document, the original authors of the instrument approved the final Spanish version (Appendix A).

#### 2.3.2. Phase 2: Validation of the NSCI in Graduated Nurses

The second phase involved the process of instrument validation in a sample of 482 qualified Spanish nurses. Participant recruitment took place from March to November 2019. The scale was sent by email to the nurses’ line managers, who forwarded it to the qualified nurses via professional email.

### 2.4. Data Analysis

The data were analyzed using IBM SPSS v22 for EFA, reliability, descriptive, and inferential analysis, while IBM AMOS v22 was used for the CFA. EFA was performed estimating the Kaiser-Meyer-Olkin (KMO) and Bartlett’s sphericity tests, extracting the loading factors (>1) using Varimax rotation. For the estimation of reliability, Cronbach’s alpha was estimated for each dimension and the entire instrument. The results for the quantitative variables were presented as mean and standard deviation (SD), while absolute and relative frequencies (n and %) were used for the qualitative variables. Mann-Whitney U test was used to determine sex differences for each dimension of the questionnaire; a parametric test was used due to size differences between groups. To analyze the goodness-of-fit of the proposed model, the indexes used were: chi-squared and degrees of freedom ratio (χ^2^/df), comparative fit index (CFI), incremental fit index (IFI) and root mean square of approximation (RMSEA).

### 2.5. Ethical Concerns

All the participants received a letter with detailed information about the aims of the study and the anonymization of the data. The research protocol was authorized by the directors of the study locations where the sample was surveyed, and it was approved by the Clinical Research Ethics Committee of Aragón (C.P.—C.I. PI18/339) prior to data collection.

## 3. Results

### 3.1. Sample Description

A total of 559 graduate nurses completed the questionnaire, but 76 were excluded due to being incomplete. The final sample comprised 483 graduated nurses, 429 of whom were women (88.8%) and 54 were men (11.2%). The mean age was 43.87 years old (SD: 10.79). 390 (80.7%) nurses were working at hospitals, while 93 (19.3%) worked in community care. They had been working for an average of 18.22 years (SD: 11.84), but had spent 5.63 years (SD: 8.08) in their current service (Table 1).

### 3.2. Exploratory Factor Analysis

Exploratory factor analysis (EFA) was performed to determine the structure of the instrument, obtaining a Kaiser-Meyer-Olkin (KMO) = 0.829 and a significant Bartlett’s test (χ^2^(91) = 2029.01, *p* < 0.001). Applying a Varimax rotation, a four factors structure was confirmed, while the item’s distribution was different from the original English version (Table 2), explaining 73.60% of the variance.

### 3.3. Reliability Analysis

The total Cronbach’s alpha value for the complete instrument was 0.84. The reliability of each item was tested using the alpha value if the item was deleted, with no benefit from the elimination of any of the items (alpha value if item deleted ranging from 0.81 to 0.84). The alpha values estimated using the whole sample ranged from 0.77 for the Perception of Capabilities dimension to 0.92 for the Leadership dimension (Table 2).

### 3.4. Confirmatory Factor Analysis

Due to the differences between the Spanish version (hereinafter referred to as NSCI-S) and the original version of the instrument in item-dimension distribution, three different structural equation models (based on the original version in English, the Taiwanese model and the EFA proposal) were tested in the Spanish sample using the CFA. The four-factor model obtained from EFA reported the best fit, being the only one with, CFI and IFI higher than 0.9 and RMSEA lower than 0.09 (Table 3).

**Figure 1:**
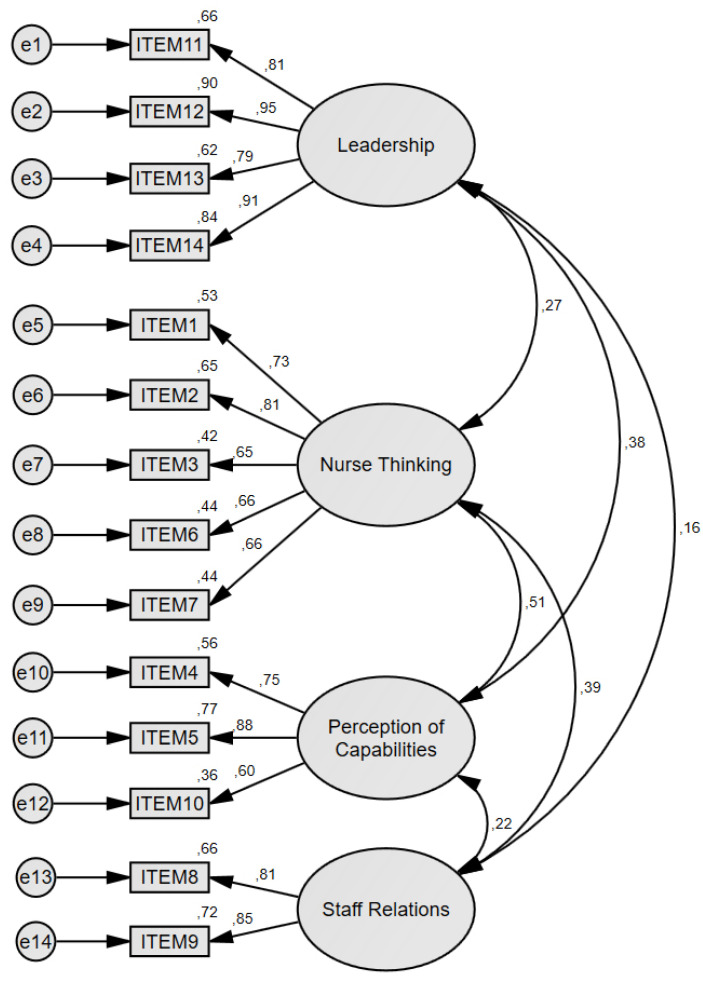
Four-factor model and item distribution for the NSCI-S.

Based on these findings, the dimensions with redistributed items were renamed to Nurse Thinking (items 1, 2, 3, 6 and 7) and Perception of Capabilities (items 5, 6 and 10), maintaining the names for the Staff Relations (items 9 and 10) and Leadership (items 11 to 14) dimensions (Figure 1).

### 3.5. Cross-Sectional Analysis

Mann-Whitney’s U test was applied to determine the differences between gender (Table 4), obtaining a significantly higher mean in the women group for the Nurse Thinking dimension (*p* < 0.001) and the Perception of Capabilities dimension (*p* < 0.05). Meanwhile, the women group reported a significantly lower mean in the Leadership dimension (*p* < 0.001).

## 4. Discussion

This study aimed to translate, culturally adapt, and validate the NSCI-S to measure nurses’ self-concept in Spain. Our findings demonstrate that the NSCI-S exhibits good reliability and validity in qualified Spanish nurses, although the distribution of the dimensions differed from that of the original tool.

The proposed model for the NSCI-S (Figure 1) was the only model reporting acceptable indexes of fit. According to Hu & Bentler [35], CFI and IFI values above 0.90 are acceptable, while Marsh et al. [36] consider values between 0.05 and 0.09 to be acceptable for RMSEA. Although the chi-square test is not considered to be a decisive index because of its sensitivity to the sample size, it is reported to calculate the chi-square/degrees of freedom, considering good fit values lower than 5 and excellent fit when the value is under 3 [37]. While the original dimensions’ distribution [1] is the most logical semantically, the questionnaire also obtained a different factorial distribution in a Taiwanese sample [32]. These differences could be explained by the different realities of nursing between countries, and also by the main cultural differences between countries. On the one hand, while in Australia there is a shortage of nurses, which leads universities to increase international enrollment in their local programs [38], in Spain there is an excess of nurses for the existing work market, forcing some of them to move abroad [29]. On the other hand, the main cultural differences between countries could also be responsible for the different distribution of items, as the impact of the cultural differences over the construction of the nurses’ self-concept has been observed before [28]. Chiefly, whilst Australian society has higher indexes of individualism, masculinity and indulgence, Spanish society has a worse tolerance to uncertainty, a higher long-term orientation and a better degree of acceptance of hierarchy [39]. Therefore, the adequacy of this model cannot be directly extrapolated to the construction of professional self-concept in all Spanish-speaking nurses, due to the different social environments and the differences in the Nursing training programs across the Spanish-speaking countries [40].

### 4.1. Renaming the Factors

Factor 2 collects items 1 to 3 from the original Care dimension, and items 6 and 7 from the Knowledge dimension. This dimension in the original questionnaire is divided into 2 in the Spanish model. Items 1 to 3 refer to how the nurse lives the experience of caring for patients, while items 6 and 7 can be understood as the enjoyment of having the necessary knowledge to develop their profession [1]. As the common point between them would be the self-experience of being a nurse, the factor was renamed Nurse Thinking since it shows how the person “is” a nurse.

Factor 3 includes items 4, 5 and 10. The first two are part of the Knowledge dimension in the original questionnaire and refer to the acquisition and application of nursing knowledge. Meanwhile, item 10 belongs to the Relationships dimension and is about helping colleagues. In the work environment, this help would be translated into helping coworkers who are in trouble with their performance. Then, the person would have to resort to their knowledge and capacities as a nurse. As the three items share the meaning of “being good/capable”, and the know-how is linked to professional identity [41], the dimension has been called Perception of capabilities.

### 4.2. Gender Differences

The gender gap observed in the Nurse Thinking and the Perception of Capabilities dimensions are in line with the higher results in professional attributes in women reported by Çöplü & Tekinsoy Kartın [9]. These differences could be explained by the maintenance of gender prejudices, supported by the stereotyped assumption that, in general, men are less sensitive and less willing to engage in caring activities [10]. Moreover, these gender differences can also be perceived in professional values [42]. These prejudices increase the risk for men entering into nursing studies of being labelled and stereotyped [43], and even leading the male students to leave their studies [44].

Also, the role tension generated by gender bias makes male students lean towards administrative positions in healthcare organizations after graduation [45]. This gender bias in nurses’ perceived leadership has been observed across the years and in different countries, affecting the leadership perception and also the relationship patterns between managers and their workforces [11,12,46,47]. In the specific case of Spanish nurses, although gender distribution is composed of 85% of women and 15% of men, approximately half of the nursing leadership positions are occupied by men [48]. Moreover, female nurses showed more conformity with the prevailing gender norms in the Spanish context [49], helping the maintenance of this gap.

### 4.3. Limitations

The study has several limitations. The first one is related to the cross-sectional design, which prevents a time-related analysis of the factors involved in the positive or negative development of the professional self-concept. Moreover, the differences obtained in the distribution of the items prevent us from establishing a direct comparison with the results obtained in previous studies using the same tool. In addition, whilst our sample was representative of the male-female distribution of the population of Spanish nurses, the total number of male participants was small, which may have introduced a degree of bias in the results. Finally, the sample was composed of clinical nurses only, excluding nurses working in research institutes and universities, and engaged in managerial positions.

## 5. Conclusions

The Spanish version of the Nurses Self-Concept Instrument (NSCI-S) has shown adequate measuring properties. The item-factor loadings obtained in this study were significantly different from the original version, leading to the proposal of a different model of nurses’ self-concept construction in the Spanish environment. The four dimensions obtained in the Spanish model had good internal consistency values, while the gender differences will require longitudinal studies or randomized controlled trials to determine the best moments to incorporate tailored activities oriented to the improvement of each dimension.

The findings regarding the different distribution of the items from the original instrument should lead future research into the analysis of different professional and social perspectives of nurses worldwide and the impact of these environmental factors on the construction and development of the nurses’ professional self-concept in different countries.

## Figures and Tables

**Table 1:** Descriptive analysis of the socio-demographic data.

	N	%
Sex	-	-
Man	54	11.2
Woman	429	88.8
Current job	-	-
Hospitalization	390	80.7
Primary Care	93	19.3
	Mean	SD
Age	43.87	10.79
Years Nursing	18.22	11.84
Years in job	5.63	8.08

**Table 2:** Rotated component matrix.

Item	Components ^1^			
	Leadership	Nurse Thinking	Perception of Capabilities	Staff Relations
NSCI11	0.83	-	-	-
NSCI12	0.94	-	-	-
NSCI13	0.84	-	-	-
NSCI14	0.92	-	-	-
NSCI1	-	0.75	-	-
NSCI2	-	0.79	-	-
NSCI3	-	0.71	-	-
NSCI6	-	0.74	-	-
NSCI7	-	0.74	-	-
NSCI4	-	-	0.77	-
NSCI5	-	-	0.84	-
NSCI10	-	-	0.73	-
NSCI8	-	-	-	0.88
NSCI9	-	-	-	0.89
Explained variance	23.52	21.46	15.01	13.14
Cronbach’s alpha	0.92	0.81	0.77	0.82

^1^ Varimax rotation.

**Table 3:** Model fits for the different NSCI models.

Model	χ^2^	gl	χ^2^/gL	CFI	IFI	RMSEA
NSCI—Spanish model	193.726	71	2.729	0.921	0.922	0.088
NSCI—Original model	329.961	71	4.647	0.834	0.836	0.128
NSCI—Taiwanese model	461.204	76	6.068	0.752	0.755	0.150

**Table 4:** Gender differences per group ^1^.

Dimension	Gender	N	Mean (SD)	Z	*p*
Nurse Thinking	Man	54	7.04 (0.75)	5.53	<0.001
	Woman	429	7.56 (0.51)		
Perception of Capabilities	Man	54	6.64 (0.84)	2.01	<0.05
	Woman	429	6.89 (0.76)		
Staff Relations	Man	54	7.28 (1.05)	0.18	0.86
	Woman	429	7.35 (0.85)		
Leadership	Man	54	6.62 (1.88)	5.91	<0.001
	Woman	429	5.42 (1.53)		

^1^ Mann-Whitney’s U test.

## Data Availability

Please contact the first author.

## Appendix A. NSCI-S

Por favor, indique su grado de acuerdo o desacuerdo con cada una de las siguientes afirmaciones utilizando la siguiente escala de respuesta.

1		8
Totalmente Falso		Totalmente cierto
1. Me preocupo por las necesidades de mis pacientes.		1 2 3 4 5 6 7 8
2. La manera en que cuido a mis pacientes me hace sentir orgulloso/a		1 2 3 4 5 6 7 8
3. Disfruto mucho cuidando a mis pacientes		1 2 3 4 5 6 7 8
4. Soy capaz de dominar los conocimientos nuevos de la enfermería		1 2 3 4 5 6 7 8
5. Soy bueno/a aplicando mis conocimientos de enfermería a la atención del paciente		1 2 3 4 5 6 7 8
6. Encuentro estimulante aprender nuevos conocimientos de enfermería		1 2 3 4 5 6 7 8
7. Me gusta tener los conocimientos para resolver problemas de enfermería		1 2 3 4 5 6 7 8
8. Me gusta trabajar con mis compañeros/as		1 2 3 4 5 6 7 8
9. Soy capaz de construir buenas relaciones laborales con mis compañeros/as		1 2 3 4 5 6 7 8
10. Soy bueno/a ayudando a mis compañeros/as		1 2 3 4 5 6 7 8
11. Soy/seré un buen líder del equipo de enfermería		1 2 3 4 5 6 7 8
12. Disfruto/disfrutaré teniendo las responsabilidades de dirigir al equipo de enfermería		1 2 3 4 5 6 7 8
13. Soy/seré un líder respetado del equipo de enfermería		1 2 3 4 5 6 7 8
14. Me gusta/me gustará liderar un equipo de enfermería		1 2 3 4 5 6 7 8
